# Blockchain based solution for secure information sharing in pharma supply chain management^☆^

**DOI:** 10.1016/j.heliyon.2024.e40273

**Published:** 2024-11-08

**Authors:** Adla Padma, Mangayarkarasi Ramaiah

**Affiliations:** School of Computer Science Engineering and Information systems, Vellore Institute of Technology, Vellore, India

**Keywords:** Ethereum, IoT, Pharma supply chain management, Secured payment, Smart contracts

## Abstract

Pharmaceutical supply chain management (PSCM) aims to alleviate logistical challenges. However, traditional online pharma systems face issues during implementation, particularly regarding transparency and fostering mutual trust among stakeholders. The primary security goals for a supply chain management (SCM) solution are ensuring authentication, confidentiality, data provenance, and auditability. The proposed blockchain-based solution (BPSCM) is implemented in three phases: registration, pharmaceutical product circulation, and secure payment. The registration phase computes the identification number upon the hashed private key along with the Edwards-curve digital signature algorithm (EdDSA) for all the stakeholders. The pharm product circulation phase implements the transactions among the participants by developing smart contracts where cryptographic operators ensure data provenance. The security analysis demonstrates that the framework effectively mitigates impersonation and collusion attacks. Performance metrics, including gas consumption, throughput, latency, and computational cost, were examined and compared to standard PSCM frameworks to evaluate the BPSCM's effectiveness.

## 1 Introduction

PSCM is a complex network of companies, people, activities, technology, information, and resources that manufacture and distribute pharmaceutical goods. Supply chain logistics entails many responsibilities, including planning, sourcing, production, delivery, and quality assurance. As the healthcare sector transitions towards custom-made items managed on an order-by-order basis, the role of statistical data mining in enhancing supply chain activities, strategic planning, design, execution, and personnel management becomes increasingly crucial [1,2]. According to the Health Research Funding Organization, 30 % of pharmaceuticals sold in underdeveloped nations are counterfeit. According to a recent World Health Organization (WHO) study, children are the most frequent victims of counterfeit medications, which are one of the main causes of death in developing nations. Counterfeit drugs not only endanger human lives but also incur significant financial losses for the pharmaceutical industry [3]. Fake medicines have played a vital role in public health and patient safety for the last few years. There is a substantial need to develop programs to prevent and detect fake medicines, including falsely labeled, unapproved, falsified, and counterfeit [50].

Since the 1980s, stakeholder roles have significantly impacted the supply chain. In the past, stakeholders had a passive role, with goods being promoted by upstream suppliers regardless of the retailer's understanding of customer demand. However, this has changed due to rapid technological advancements, intense market competition, and sophisticated supplier networks. Stakeholders now play a crucial role in the supply chain, particularly in predicting real-time customer demand. This shift has influenced retailers' changing positions, leading to the outsourcing of non-core activities, reduction of excess inventory, and increased reliance on information technology [4].

The pharmaceutical sector is critical to protecting public health by providing life-saving pharmaceuticals to individuals worldwide. Establishing an effective and dependable supply chain is crucial. However, the present PSCM facing various issues in detecting counterfeit products, a lack of transparency, ineffective monitoring, and security flaws. These difficulties could hinder access to critical pharmaceuticals, jeopardize patient safety, and raise overall costs throughout the system. The existence of counterfeit drugs in the PSCM is a significant threat to the community's health. Creating an effective traceability system is critical for verifying the legitimacy of medications throughout their entire existence. This implies tracing the origin of materials, processing, packaging, transportation, and pharmacy delivery. In addition to counterfeiting, the pharmaceutical industry faces several significant challenges, such as the inability to verify the provenance of medicines during pandemics. The issue of uncontrolled drug prices needs to be addressed. Foreign-imported Active Pharmaceutical Ingredients (APIs) can disrupt production and mislead consumers [55]. Gupta and Namasudra [64] have presented a framework to accelerate virtual machine migration in cloud computing by reducing downtime, migration time, and data transfer rate. However, the system's performance was affected by complex master-slave coordination.

Recent technological advancements have opened up promising avenues to address these challenges. The shift from centralized cloud platforms to decentralized and distributed technology is a significant development. Blockchain offers a groundbreaking approach to supply chain tracing, utilizing a chain of blocks in chronological order managed by network participants. Cryptographic hash techniques ensure these blocks' immutability, serving as a transaction history record. With its unique features of immutability, transparency, and security, Blockchain Technology (BT) has emerged as a disruptive force in various sectors, including pharmaceuticals [52,53]. Researchers and industry experts are exploring the potential of blockchain in the PSCM, keen to leverage its benefits to overcome key challenges and create a more sustainable, efficient, and secure environment. BT in electronic SCM allows for real-time transaction recording and tracking, ensuring dependability and preventing counterfeiting.

Supply chain analysis entails assessing each economic agent's function and contribution across the supply chain, from raw material procurement to final product delivery. Developing countries require assistance in managing supply chains, which include political instability, corruption, and a restricted adoption of modern technology [6]. Information sharing across supply chain trading partners is becoming progressively crucial as the economy grows unstable. Early prediction of end-user demands can reduce future uncertainty and eliminate leftover stock. Furthermore, supply chain visibility enables managers to react efficiently to timely data and facilitates efficient planning [7]. Moreover, there is a pressing need for equitable and sustainable drug distribution, considering disease burden, availability, accessibility, and environmental sustainability. In this context, enhancing inventory management processes, implementing targeted interventions, and harnessing technologies like blockchain can significantly enhance the performance of the PSCM and improve healthcare delivery outcomes [5]. Table 1 provides a comprehensive overview of the involvement of various stakeholders in SCM, both with and without the integration of blockchain.

**Table 1:** Pharma supply chain using BT.

PSCM role players	PSCM without blockchain	Merits of blockchain
Suppliers (P_s_)	Drug raw molicules	Storage of drug raw molicules
Retailers (P_r_)	Traceability issues	Achieves traceability
Drug manufactures (P_dm_)	Drug production	Storage of drugs
Distributors (P_d_)	Lack of tracking	Decentralized tracking
IoT container (P_IoT_)	Lack of tracking	Decentralized tracking using IoT sensors
Consumers (P_c_)	Drug quality issues	Attains compelled quality drugs

To comply with regulations pertaining to the European Union General Data Protection Regulation (EUGDPR), PSCM systems must adhere to various fundamental guidelines, including consent-based data processing, which ensures that personal data is only gathered and handled with the approval of stakeholders and customers. The system must also ensure transparency and accountability by giving explicit information regarding data management. Data integrity and accuracy are critical, which means that data must be maintained correctly and securely, with safeguards against unauthorized access or deletion. Furthermore, privacy by design should be built into the system from the start, ensuring that data security is a priority throughout the development process. Data access should be limited to authorized entities, and all personal data activity must be documented. Finally, personal data should be erased once it is no longer needed. Regulatory-compliant designs ensure hashed transaction data (e.g., drug batches, expiration dates) is stored on the blockchain while sensitive data remains within secure systems to comply with regulations like GDPR.

The compatibility aspect of the proposed model with existing PSCM systems is essential to ensure seamless adoption and practical implementation. BPSCM enhances data transparency, security, and efficiency within the network without requiring a complete infrastructure overhaul. The developed system can integrate with traditional PSCM systems through APIs (REST), middleware (data integration, service-oriented), and regulatory-compliant designs. APIs allow real-time data exchange between drug tracking and order management, ensuring legacy system data is incorporated into the blockchain for provenance and transparency. Middleware translates data from traditional PSCM systems into a blockchain-compatible format.

Shankar et al. [56] presented a multi-signature schema for digital documents using the EdDSA algorithm. Signing and verification are performed using generated public and private keys. It is cost-effective and secure, and the computational cost is less than other cryptography techniques. Datta and Namasudra [59] introduce a novel blockchain-based Electronic Medical Record (EMR) sharing framework that leverages Mobile Edge Computing (MEC) and consumer electronic devices. This framework incorporates additional security layers using Advanced Encryption Standards (AES) encryption, stores encrypted EMRs and diagnosis reports on InterPlanetary File System (IPFS) and utilizes smart contracts and the Proof of Authority (PoA) consensus algorithm for efficient management and faster transactions. However, this schema needs more comprehensive detail in terms of the data input process and the potential for malicious entities to transmit irrelevant data.

Lou et al. [8] have presented a blockchain-based Supply Chain Electronic System Cooperation Framework (SESCF) to address authentication challenges and delays in capital flow, logistics, and product circulation. Radio Frequency Identification (RFID) is used for product identification to ensure the integrity of the SESCF model. Hasan et al. [49] have developed a blockchain-based model to handle challenges related to shipment by integrating IoT. Data is collected through IoT sensors and stored in blockchain-enabled databases in the cloud. Smart contracts are implemented to monitor deviation while collecting the data from sensors to implement secure payment, authentication, and product traceability. However, this model's transaction processing time is high.

Datta and Namasudra [62] have presented a blockchain-based counterfeit drug schema by adopting an IPFS server for off-chain storage and Proof of Work (PoW) for consensus verification and block validation. However, this schema fails to address the smart contract vulnerabilities. Mackey and Nayyar [50] have proposed smart contract-based models for counterfeit drugs. However, finding breaches after a customer completes payment can lead to a time-consuming process to verify and get a refund, posing a significant risk to the customer. These lead to a direct impact on customers for poor quality medicine delivery and business growth. In this case, the temperature of the containers might be crucial to prevent quality impairment and shipping contamination. Dwivedi et al. [9] have presented a blockchain-based PSCM system with reliable information sharing. The consensus process verify the authenticity of each new block and transaction, while the smart contract was employed to ensure secure cryptographic key distribution. However, intruders may compromise the security aspects of the included Certificate Authority (CA), which may again encourage external and internal adversaries to execute data tampering and collusion attacks.

Motivated by the vital significance of a realistic supply chain solution and the widespread adoption of blockchain and IoT [10] technology across numerous industries, we propose a BPSCM to connect various stakeholders in a trusted environment and promote product sharing. Despite technical hindrances, the candidate model is designed to significantly improve trust, efficiency, coordination, and transparency, providing a clear view of the entire supply chain process. The IoT sensors are implemented in the containers to facilitate product traceability and ensure product quality. They detect and report temperature deviations in the smart contract, notifying all stakeholders of the potential impact on the integrity of medical products. BT is used to accomplish the security features of secrecy and authenticity, achieved using symmetric and EdDSA algorithms. Smart contracts regulate interactions between multiple stakeholders to efficiently guarantee high-end customer satisfaction without involving intermediates. An energy-efficient Proof of Stake (PoS) consensus protocol was utilized for transaction validation and block mining process. Another challenge associated with the PSCM is ensuring product integrity. To implement this, the proposed BPSCM smart contract has been designed in such a way as to continuously monitor the product quality throughout the life cycle and raise alerts to appropriate stakeholders about the deviation incurred in maintaining temperature to prevent product contamination. The major contributions of this work are as follows.

1. An Ethereum smart contract-based model is proposed to ensure data provenance, transparency and integrity of the BPSCM framework.
2. Decentralized storage IPFS and EdDSA are used to store transactions and achieve confidentiality, scalability, high transaction throughput, and reduced computational cost.
3. Phases of the presented framework registration, authentication, and secure payment were implemented through deterministic Smart contracts. PoS consensus has been used to accelerate block mining and transaction validation.
4. The proposed smart contract was analyzed against security vulnerabilities using Oyente and the MyThril tool. The performance analysis of BPSCM performs better than the existing models.

The remainder of this paper is organized as follows: Section 2 reviews existing models' key contributions and limitations. Section 3 provides background on our proposed model, including entities and security goals. Section 4 discusses the proposed BPSCM and its corresponding smart contract algorithms. Section 5 presents the testing and validation, and security analysis. Section 6 shows the performance analysis of the proposed model. Finally, Section 7 concludes the paper.

## 2 Related works

The automated supply chain faces technical hindrances in ensuring consistency in maintaining the product's state among the diverse stakeholders. Blockchain is an efficient tool that facilitates transparent product state implementation. Preventing fake product entry for the candidate BPSCM is vital. Hence, this section analyzes the various blockchain-enabled supply chain frameworks that mitigate the breaches encountered through counterfeit products and other challenges. Mishra et al. [11] introduced an efficient blockchain-based framework that utilizes smart contracts. This innovative approach ensures secure and transparent tracking of pharmaceutical records. The framework chunks the data for efficient search to implement parallel searching. However, this model is appropriate for small-scale networks, even if it performs best.

Sharma et al. [63] proposed a secure cloud storage system integrating BT. This schema utilizes smart contracts, encryption, and optimization techniques to identify errors and code regeneration. However, the computational overhead of blockchain operations affected the system's performance. Namasudra and Sharma [65] proposed a blockchain-based secure cab-sharing system. This schema employs re-encryption, delegated proof of stake consensus, and a reputation system to ensure security and privacy. However, the system fails to address the authentication services.

Yazdinejad et al. [66] have presented an energy-efficient software-defined networking (SDN) architecture for IoT networks using blockchain. This framework used a cluster structure with a new routing protocol for secure access control. However, the system performance is higher throughput, lower delay, and lower energy consumption compared to traditional methods. Yazdinejad et al. [67] present a novel anomaly detection framework for blockchain-based IIoT networks in smart factories. This schema utilizes a cluster-based architecture, federated learning, and various machine learning models to improve efficiency and detect anomalies. Additionally, the effectiveness of the anomaly detection models may vary depending on the specific characteristics of the IIoT network. Yazdinejad et al. [68] proposed a secure, intelligent fuzzy blockchain framework for network attack detection in IoT environments. The framework incorporates a fuzzy deep learning model, fuzzy control systems, metaheuristic algorithms for optimization, and fuzzy matching for fraud detection. Evaluation results demonstrate its threat detection and decision-making effectiveness within blockchain-based IoT networks.

Yazdinejad et al. [69] have presented an Auditable Privacy-Preserving Federated Learning (AP2FL) framework for medical devices. AP2FL uses a trusted execution environment to prevent data leakage during training and aggregation. Active Personalized Federated Learning (ActPerFL) and Batch Normalization (BN) were used to aggregate user updates and identify data similarities for non-IID data. Yazdinejad et al. [70] have proposed a hybrid privacy-preserving federated learning framework tailored for Next-Generation Internet of Things (NG-IoT) environments. This framework employed two-trapdoor homomorphic encryption and server protocol to mitigate the impact of irregular users, while the asynchronous hybrid algorithm reduced user dropout rates. Performance evaluations demonstrated the framework's superiority over existing solutions in terms of functionality, accuracy, and reduced system overheads.

One of the most critical issues facing the pharmaceutical sector is the detection of counterfeit products, which poses substantial risks to consumers and the environment. Jha et al. [12] introduced a Hyperledger Fabric (HLF) based blockchain solution in this context. This groundbreaking framework, consisting of ledger, smart contract, and user interface modules, holds immense potential to bolster the safety of pharmaceutical products by effectively identifying counterfeit drugs. However, it is crucial to note that this solution is primarily designed to track falsified medication supplied within authorized supply networks. Al-Farsi et al. [13] focused on addressing the internal and external attacks on the entities involved in SCM. It provides transparent interaction between participant entities by allowing business rules. These dynamic rules protect the system from internal and external attacks. However, it handles safe and unsafe constraints instead of valid constraints.

Ma et al. [14] proposed vulnerability detection on transactions using a hierarchical graph attention network. They utilized the Abstract Syntax Tree (AST) and control flow graph to analyze the smart contract functions. Since the flow of the transactions is precisely recorded through the AST and control flow graph, the model can detect vulnerabilities more quickly and precisely than the other detection models. Subramanian et al. [15] proposed that the NEM blockchain alleviates the burden of tracking drugs using mobile applications. The presented model uses NEM cryptocurrency and QR codes to label pharmaceutical products. Physicians, patients, and pharmacists have implemented product authenticity. Proof of Import (PoI) is used in smart contracts to validate the products. However, it does not include a direct drug monitoring system for doctors and patients. Similarly, Konapure and Nawale [16] developed a blockchain-based solution to enhance the traceability of pharmaceutical products through supply chain applications. Smart contracts are intended to reduce third-party involvement requirements.

In the traditional pharmaceutical supply chain, blind parties lead to information fragmentation, diminished participant responsibility, incomplete information at every level, and the potential for introducing counterfeit medications. These could endanger patients and result in financial loss. Fragmentation can lead to inaccurate demand estimates, which could harm consumers and even have fatal effects. To eliminate blind parties and data fragmentation between involved parties, Bapatla et al. [17] proposed an efficient smart contract-enabled PharmaChain model. Smart contracts and Proof of Concept (PoC) address access control policies and scalability issues. Regarding resilience against cyber-attacks, re-entrance attacks, and randomness by using the Oracle model.

Uddine [18] proposed a Medledger framework to identify phony medicine in the pharmaceutical ecosystem. The framework uses the HLF to ensure stakeholders' validation, authentication, and verification. Chain code exchanges critical information between participating entities over fine-grained access control for drug traceability. The Medledger network validates and tracks the activity of stakeholders using the smart contract modules drug registration, consignment accumulation, and transaction update contracts. Agrawal et al. [19] implement forward and backward supply chain models to minimize the delay and cost incurred by the drug delivery process. Utilizing Hyperledger Composer while modeling the assets and maintaining the transaction history greatly enhances security, transparency, and efficiency.

Similarly, Musamih et al. [20] proposed a blockchain-based solution by leveraging the Ethereum platform's smart contracts and encryption techniques to improve healthcare management's accuracy and transparency. This schema ensures authenticity by offering a combination of identity management and membership services. Streamlines processes and gives transparent, secure drug monitoring to various stakeholders, including manufacturers and patients. This innovative method has the potential to transform medication traceability and eventually improve security and patient safety across the entire healthcare supply chain, even though there are obstacles such as regulation and scalability. Chiacchio et al. [21] introduced a solution using Non-Fungible Tokens (NFTs) to enhance PSCM tracking and tracing capabilities. NFTs create a unique identifier for each parcel assembled by the packaging lines of a pharmaceutical factory. The NFTs are characterized by the Owner's Public Address to the manufacturer, making it impossible for others to append information to the smart contract. By minting NFTs, the system inherits the benefits of BT, offering enhanced transparency and security. This framework deployed in the VeChain Thor blockchain test network using PoA to guarantee enhanced scalability.

To mitigate the problems caused by counterfeit pharmaceuticals, Abbas et al. [22] presented a blockchain model to implement transparency and drug recommendation systems for consumers. The blockchain module tracks drug movement through the supply chain, ensuring authenticity and transparency. The machine learning module uses reviews to recommend suitable medications to consumers. This combination is facilitated through HLF and REST API between both models to enhance the accuracy and efficiency of text classification models. Gaur et al. [23] proposed a blockchain and machine learning-based framework for effective data processing and storage. The model improves security and reduces storage consumption through Smart contracts. A support vector machine detects threats and classifies the data. Attribute-Based Encryption (ABC) schema allows access control based on attributes. Typically, it involves using a public key and a private key, where the private key is associated with specific characteristics. The private key decrypts data encrypted using the corresponding public key and attributes. CP-ABE demonstrated security against a chosen ciphertext attack. However, this model analyzes the system performance in terms of privacy, identification, and authentication.

Kaneriya and Patel [24] proposed a credential verification system for specific attributes without revealing the contents of other attributes. All the attributes are placed randomly in a tree, which leads to a complex situation where attackers can identify a particular entity. This schema addresses issues of record forgery, tampering, and time-consuming verification. However, it enhances latency, space complexity, and bandwidth. Bocek et al. [25] proposed a blockchain-based framework to secure IoT data by tracking the temperature settings of IoT-enabled containers. This process reduces expenses due to process optimization, automation, energy efficiency, and data-driven approaches, and data integrity and regulatory compliance are guaranteed. Blockchain attracts companies from various sectors with its ability to save costs, decentralize infrastructure, and minimize inefficiency. However, this requires handling the security of IoT sensor data within an access control system.

Rehan et al. [26] proposed an innovative HLF method with IoT sensors. These sensors provide real-time tracking, increased transparency, and secure temperature monitoring for perishable items. Distributed database through HLF promises quick transaction speeds and guarantees data integrity. Experiments indicate fast data transport (data compression, efficient routing algorithms, and optimized network protocols), block formation (grouping transactions into blocks, consensus algorithms, and block size management strategies), and network connectivity (peer-to-peer communication), with transaction times as low as 48 s. However, this model is a viable solution for safe, transparent, and efficient supply chain management, especially for temperature-sensitive items. Due to intricately supply networks, consumers frequently need information about pharmaceutical items. Aslam et al. [27] proposed a framework to improve customer trust and access information on the Ethereum blockchain. This schema improves efficiency and traceability by using smart contracts to track medications along the supply chain. However, more investigation is required for broader implementation of the real-time pharmaceutical industry. Table 2 highlights the blockchain-based approach's contributions to improving security and transparency in the pharmaceutical supply chain.

**Table 2:** Review of traditional supply chain management using smart contracts.

Ref	Key observation	Domain	Implemented platform	Limitation	Metrics
[28]	Proposed framework utilizes a layered approach built on disruptive technologies to streamline logistics within the agricultural sector. Automation encompasses tasks within individual companies (intra) and between companies (inter). The proposal aims to minimize gas fees through a creative combination of different approaches.	Agriculture	Ganache, Truffle suite, Solidity	Test cases regarding consumer refunds and prompt delivery are yet to be considered.	Gas cost
[29]	The candidate framework mitigates food quality and payment issues through QR and bar codes.	Food SCM	DApp, HLF	An implementation needs more resources, which in turn leads to high costs.	Transaction time
[30]	BLS signature ensures authentication.	SCM	Ethereum	Further research is required to utilize a wider variety of blockchain structural properties to maximize efficiency within SCM.	Gas cost, Computation cost
	Data Provenance details can be accessed without accessing the original content.				
	Smart contracts are designed to be robust against collusion attacks, impersonation attacks, and tampering attacks.				
[31]	Proposed online pharma SCM. The model efficiently detects breaches during transportation using sensors, and payment is refunded to customers.	PSCM	Ethereum	The role of drug manufacturers, distributors, and consumers may be considered.	Execution cost, Transaction cost
[32]	Proof of delivery for physical assets has been adopted to reduce disputes in automatic payments involving single or multiple transporters. The model effectively prevents replay, denial-of-service, and man-in-the-middle attacks.	Transportation	Ethereum	Prototype design is required to evaluate secure and automated digital asset sale transactions.	Execution cost, Transaction cost
[33]	Proposed resource sharing for supply chain management based on smart contracts ensures reliability and data authenticity in supply networks and efficient resource utilization in networks with large outsourcing and production surpluses.	SCM	Ethereum	Visibility and accessibility for specific actors should be determined and separated.	Computational cost
[35]	An ESP32S2 device is used as a proof of concept to assess and confirm the proposed data storage protocol's functionality. A smart contract and a decentralized web application are intended to display and demonstrate sensor data gathered from the public blockchain.	SCM	PoC, Mythix	Challenges raised by stakeholders must be addressed. Enhanced security analysis is required.	Computation cost, Power consumption, Memory usage
[36]	Proposed a decentralized authentication framework for smart city. The ECC algorithm generates the key, and security goals are ensured through trust and authorization smart contracts.	SCM	Contiki, Multichain, COOJA	No discussion about cost analysis and vulnerability analysis.	Throughput, Access time Delay, Execution time
[37]	Proposed a supply inventory system based on blockchain that uses trusted environments and smart contracts. Large supply chains with several complicated transactions benefit from increased transparency and vendor coordination.	SCM	Ethereum	No discussion on security analysis.	Gas cost
[59]	Proposed secure SCM by implementing various cryptographic techniques. These techniques ensure secure, computationally efficient, fast verification and resistance to quantum attacks. Multiple phases are involved by using different smart contract functions.	SCM	Ethereum	No discussion about cost and vulnerability analysis.	Throughput, Processing time, Latency
[62]	Proposed blockchain-based counterfeit drug scheme utilizes IPFS for off-chain storage and PoW for consensus verification and block validation.	PSCM	Ethereum	No discussion about smart contract vulnerabilities.	Communication cost, Throughput, Computation cost

Globalization amplifies problems in supply chains, such as supplier relationships, pricing, product authenticity, transparency, and customer service. All these factors need careful consideration. The literature reviewed in this section highlights the importance of privacy aspects and stakeholder inclusion for ensuring reliable supply chain services. While existing frameworks analyzed here utilize cryptographic techniques and smart contracts to achieve authentication and transparency, a compelling need remains for a simpler, more cyber-resilient automated supply chain framework.

## 3 System model

**Fig. 1:**
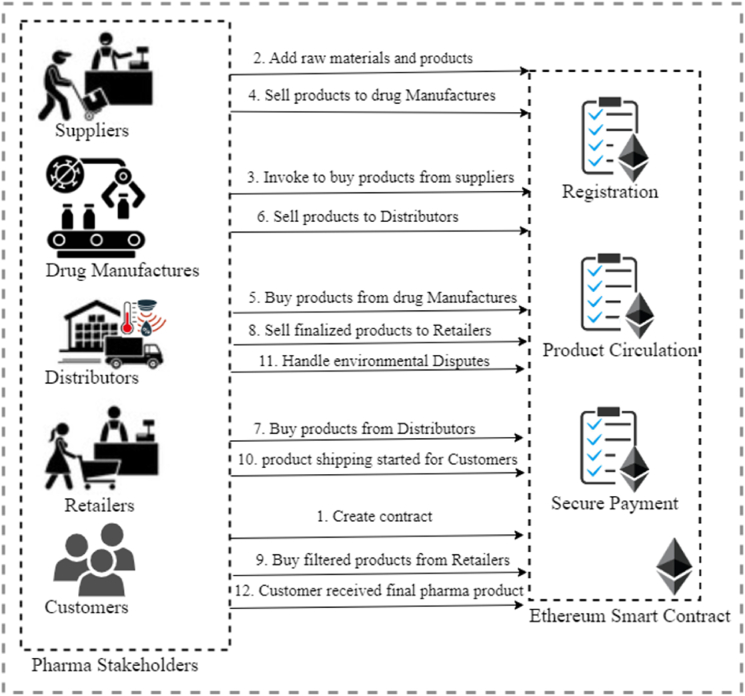
Real world entities and workflow of the presented BPSCM system.

This section presents the decentralized PSCM solution built upon the Ethereum platform blockchain to ensure authentication, product traceability, and product provenance. Fig. 1 describes the proposed model BPSCM solution along with its diverse stakeholders. To mitigate the technical difficulties incurred in logistics of the presented BPSCM includes suppliers ($P_{s}$), retailers ($P_{r}$), drug manufactures ($P_{dm}$), distributors ($P_{d}$), IoT containers ($P_{IoT}$), and consumers ($P_{c}$), such that entity identification is established through Ethereum account address. The description of the various building blocks of candidate BPSCM is summarized as follows.

➢ Suppliers ($P_{s}$): Companies act as suppliers, collecting raw materials and supplying them to drug manufactures.
➢ Drug manufactures ($P_{dm}$): Drug manufactures are companies that produce pharmaceutical drugs. They are responsible for formulating novel pharmaceutical compounds, engaging in large-scale production, and conducting comprehensive assessments to determine their safety and effectiveness.
➢ Distributors ($P_{d}$): Distributors sell the products to retailers. They often store the products in warehouses before shipping them to retailers.
➢ Retailers ($P_{r}$): After receiving the product from a drug manufacturer or distributor, retailers set reasonable pricing policies and sell the product to consumers.
➢ IoT container ($P_{IoT}$): Pharma products are delivered through containers equipped with IoT sensors to track and trace the product provenance record (delivery). IoT containers are equipped with sensors (temperature sensor, pressure sensor, GPS tracker) to track the temperature of pharma products, pressure due to accident jerk or opening of the container, and location of the container [25,31].
➢ Customer ($P_{c}$): Customers purchase the products provided by the retailers; then, legitimate customers pay for products at a specific price if there are no product violations or damage. In BPSCM, customers can check the validity of products in the blockchain.
➢ IPFS server: All product details hash codes are uploaded to the IPFS server to reduce the blockchain's storage burden.

### 3.1 Threat model

The objective of an adversary seeking unauthorized access is to disrupt or monitor the distribution of pharmaceutical items. Adversaries are part of customers in the supply chain, and they may collude with the IPFS server to access product details stored in the server. Once the adversary gains access to this information, it could be altered and used for other purposes. Adversaries attempt to obtain access to the SCM by posing as legitimate users. Once they have access, they try to learn as much about the system's operations as possible. It would capture the product provenance record and supply chain [9,13].

### 3.2 Security goals

Our meticulously designed BPSCM model withstands attacks and fulfils crucial security and privacy objectives.

➢ Integrity and confidentiality: In the BPSCM model, all stakeholders uphold a product provenance record through a hash of their identity and password. The use of EdDSA to generate public and private keys ensures authentication and integrity. Product provenance is implemented to be resilient against various attacks such as forgery, tampering, and impersonation.
➢ Secured payment: Ensuring secure payment for stakeholders is a pivotal and often challenging aspect of the SCM system. In the BPSCM model, stakeholders can pay for products after verifying a proper authentication key generated using keccak256. Deploying a smart contract on the blockchain adds more security to this process.
➢ Record Auditability: In BPSCM, IoT sensors are installed within the container to track products. The other stakeholders know the product's shipping status without accessing the product's contents. This ensures that a secure hash algorithm and a Merkle tree storage structure for blockchain are used.

Our proposed system ensures privacy aspects of the product's provenance record, allowing anyone to verify its authenticity without access to its contents. It also enables stakeholders to track the product's journey through the supply chain tracking system and guarantees that all parties get compensated. Through this impressive set of security goals, our suggested approach can significantly enhance supply chain security. Table 3 outlines the deployed smart contract functions and their key characteristics.

**Table 3:** Smart contract functions intend to various stakeholders along with their privileges.

Function	Input	Output	Stakeholder involved	Description
Constructor()	NA	True/False	P_r_, P_dm_, P_s_	Initialize the stakeholders' Ethereum account addresses, parameters, states, and IPFS hash.
product_Add()	product_Name, product_Id, product_Quantity and product_Price	True/False	P_r_, P_dm_, P_s_, P_c_	Add products/items on the BPSCM.
product_Remove()	product_Id	True/False	P_r_, P_dm_, P_s_, P_c_	Remove the item from BPSCM
getnumberof_Products()	NA	Integer number	P_r_, P_dm_, P_s_, P_c_, P_d_, P_IoT_	Return the number of items ordered on request.
get_Product()	product_Id	Entity/object	P_r_, P_d_, P_IoT_	Returns information regarding the item for authentication.
order_Placed()	Order_Id, product_Id, product_Quantity, customer address	True/False	P_r_, P_dm_, P_s_, P_c_, P_d_	Orders are placed based on consumer requests, and other stakeholders are also indirectly involved.
is_Orderfullfilled()	product_Id	True/False	P_r_, P_dm_, P_s_, P_c_, P_d_	Returns sufficient items are their to full fill the order. If an order has already been placed, it returns false; otherwise, it returns true by checking the availability of items.
wholesaler_Add() and wholesaler_Remove()	Wholesaler address	True/False	P_d_	Based on the request, the number of wholesalers will be added and removed on BPSCM.
retailer_Add() and retailer_Remove()	Retailer address	True/False	P_r_, P_d_	returns number of retailers will added and removed on BPSCM based on the request.
pharma_MAdd() and pharma_MRemove()	Pharma manufacture address	True/False	P_dm_, P_d_	Add the pharma manufacture and remove based on request on BPSCM
quality_Control()	Address of quality control	True/False	P_dm_	Check the quality of the product and return true/false accordingly.
env_Datarecord()	Container_Id, temperature, humidity, timestamp	Entity/object	P_IoT_	Record the environmental change data in IoT container.
location_Record()	Container_Id, latitude, longitude, timestamp	Entity/object	P_IoT_	Record the location details of an authorized entity.
payment_Order()	order_Id, product_Id, product_Quantity, product_Price, supplier address, and customer address	True/False	P_r_, P_dm_, P_s_, P_c_, P_d_	A payment order was generated for a customer for product delivery.
deliver_Order()	order_Id	True/False	P_r_, P_c_	Return order is delivered or not.
order_Dispute()	order_Id	String	P_r_, P_dm_, P_s_, P_c_, P_d_	Returns the string value to indicate the order dispute.
refund_Processed()	order_Id, amount	Entity/object	P_r_, P_dm_, P_s_, P_c_, P_d_	The refund status was generated and received by a customer.
change_Ownership()	Ethereum address of new owner and old owner	True/False	P_r_, P_dm_, P_s_, P_d_	Current owner has changed, and this function is accessible to the current stakeholder owner.
update_Stakeholder()	address	Integer number	P_r_, P_dm_, P_s_, P_c_, P_d_, P_IoT_	Update the stakeholder address to avoid the already existing account address.
upload_IPFShash()	item_Id, IPFS hash	hash	NA	Item hash value uploaded for auditing purposes.
get_Trackrecord()	trace_Id	Entity/object	P_r_, P_c_, P_IoT_	Get the tracking information about the product delivery.

## 4 Proposed BPSCM

This section presents the details of the proposed BPSCM, including various phases, such as registration of all stakeholders ($P_{s},P_{r}$, $P_{dm}$ $P_{d}$ $P_{IoT}$, $P_{c}$), circulation of pharma products (product services, quality control and maintenance), and secured payment between stakeholders. The proposed framework maintains the pharma product information among the diverse stakeholders to ensure the appropriateness in delivering the final product to the end user. Here, we mainly focus on maintaining the confidentiality of the product's provenance record and preventing its information from being modified illegally throughout its circulation using symmetric encryption and EdDSA. EdDSA is faster, more secure against attacks, and requires less processing time than other cryptographic algorithms. The flow of the presented model is as follows.

➢ Initially, every stakeholder undergoes a registration process, and their identities are broadcast to other entities (Algorithm1).
➢ Pharma product details are encrypted using symmetric encryption, and the product provenance record is verified through the EdDSA technique.
➢ Encrypted pharma product details are shared among stakeholders (Algorithms 2 to 5), and environmental deviations are alerted to the appropriate entity.
➢ After product delivery, payment is established among stakeholders (Algorithm 6)
➢ All the transactions are validated and added to the blockchain using PoS Consensus.

EdDSA is a variant of the Schnorr signature method based on Elliptic Curve Cryptography (ECC). Ed25519 and Ed448 are variations of EdDSA based on Edwards curves. The experiment considered Ed25519, which generates keys using Curve25519 and SHA-512. This algorithm develops a key pair of 256-bit keys and 512-bit hashes. These hash functions exhibit four key characteristics: one-way functions (easy to compute a message's hash but infeasible to reverse), preimage resistance (finding another message with the same hash is computationally hard), second-preimage resistance (finding another message with the same hash for a given message is computationally impossible), and strong collision resistance (finding message pairs with the same hash is infeasible). This approach is faster and more secure against several cryptographic attacks than other public key cryptographic algorithms [57,58,60].

The private key (${PRk}_{si}$) is a randomly generated hashed number during the encryption process, while the public key (${PUk}_{si}$) is derived from the private key. There are two forms of EdDSA, according to NIST and IRTF standards [60], depending on how the randomness is generated: ${\;r}_{i}={Prand(dk}_{i},M)$ in the first case and ${\;r}_{i}={Prand(dk}_{i},H\left(M\right))$ in the second. Where ${dk}_{i}$ is derived key, M is a message, Prand is a sudo random number, and H(M) is the hash of the message. In Ed25519, the ${PRk}_{si}$ is randomly generated integer is further ${PUk}_{si}$ is generated using a curve generator $G_{i}$ on an elliptic curve as shown in equation (1) and the hash calculated by following equations [56].

(1) $${PUk}_{si}\leftarrow \left({PRk}_{si}*G_{i}\right)$$

(2) $$h\;\leftarrow \{H(r_{i}+{PUk}_{si}+M)modQ\}$$

Where Q is a prime integer number

Then, the signature and ${\;r}_{i}$ is calculated by using the following equations (3), (4)

(3) $${Sig}_{i}\leftarrow \left(\mathrm{int}+h*{PRk}_{si}\right)$$

(4) $$\left\{r_{i},{Sig}_{i}\right\}\leftarrow E_{sign}\;\left(M,{PRk}_{si}\right)$$

After generating the signature, the public key is used for verification. First, calculate the hash using equation (2), then calculate the verification points $\left\{P_{1},P_{2}\right\}$ using equations (5), (6)), and finally verify that both verification points are equal $\left(P_{1}=P_{2}\right)$. The verification of the signature is represented by equation (7).

(5) $$P_{1}\leftarrow \left({Sig}_{i},G_{i}\right)$$

(6) $$P_{2}\leftarrow \left(r_{i}+h*{PUk}_{si}\right)$$

(7) $$\frac{\text{Valid}}{\text{Invalid}}\leftarrow E_{verify}\left(M,{PUk}_{si},r_{i},{Sig}_{i}\right)$$

In BPSCM the involved stakeholders are $P_{s},P_{r}$, $P_{dm}$ $P_{d}$ $P_{IoT}$, $P_{c}$, and IPFS server. These stakeholders are represented as $S_{i}$, where iϵ1,2,3,4,5,6 and every stakeholder maintains the unique Ethereum account address (${EA}_{si}$) along with ${PRk}_{si}$ and ${PUk}_{si}$ which are generated using EdDSA. The encrypted pharmaceutical details are initially stored in an IPFS server to preserve privacy and confidentiality.

### 4.1 Registration smart contract

In BPSCM, every stakeholder $S_{i}$ maintains separate unique ${EA}_{si}$ along with ${PRk}_{si}$ and ${PUk}_{si}$ to achieve secure authentication using smart contracts. Additionally, stakeholder $S_{i}$ has to submit own password (${Pwd}_{si}$), which is rendered using a random verification number and a hash of the private key when submitting the registration contract. And generated identity ${Id}_{si}$ in equation (8) has broadcast to all stakeholders to resist the password guessing attack and the private information of stakeholders $S_{i}$ will not be disclosed anywhere.

(8) $${Id}_{si}=S\_Register\left({Pwd}_{si},h\left({PRk}_{si}\right)\right)$$

**Table:** 

Algorithm 1: Stakeholders Registration
*Input*: ${Pwd}_{si}$, ${PRk}_{si}$
*Output*: Identity ${Id}_{si}$ of stakeholder
*Procedure*: Create a registration smart contract
Generate Ethereum address ${EA}_{si}$ for all stakeholders: ${EA}_{si}\;\leftarrow \;S_{i}$
${S_{i}:S_{i}\leftarrow Pwd}_{si}$, where iϵ1,2,3,4,5
${Id}_{si}=S\_Register\left({Pwd}_{si},h\left({PRk}_{si}\right)\right)$
Broadcast ${Id}_{i}$ to all $S_{i}$
State: state ← created
Emit an event successfully generates identity ${Id}_{i}$ for all stakeholders $S_{i}$
*EndProcedure*

### 4.2 Circulation of pharma products smart contract

**Fig. 2:**
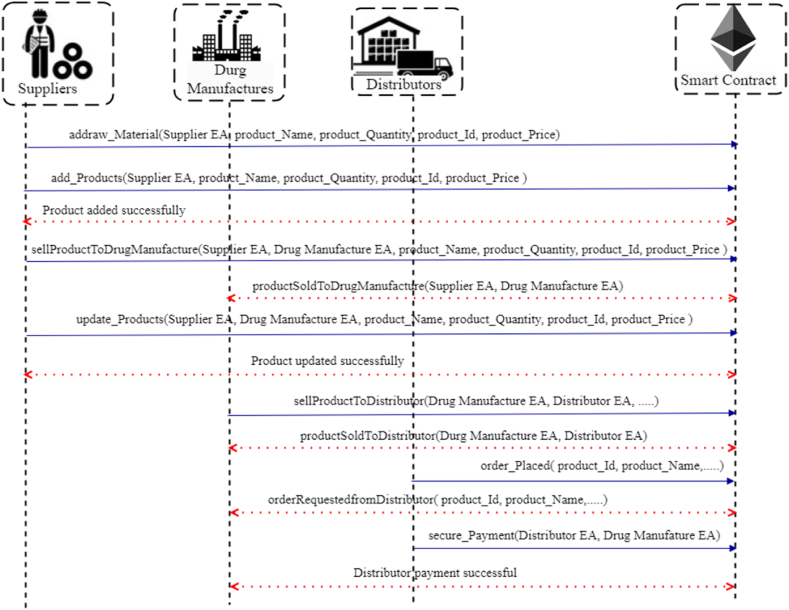
Sequence diagram showing the interaction between supplier, drug manufactures, distributor and smart contract.

In BPSCM, product information is shared with different stakeholders. We used the EdDSA signature schema and symmetric encryption to verify the product provenance record during pharmaceutical product circulation. This ensures the traceability of the product and protects it from unauthorized modifications by malicious entities. Fig. 2 illustrates the sequence flow of product circulation between suppliers, drug manufacturers, and distributors.

Case1 Supplier to Drug manufactures

Suppliers provide the raw materials API to drug manufactures, they took the responsibility of generating the product provenance record R1, and it represented as equation 9

(9) $$R_{1}\;\leftarrow \;{{Id}_{s1}⊕Id}_{m}⊕{Ai}_{m1}$$

Where ${Id}_{s1}$ represents the identity of suppliers, ${Id}_{m1}$ specifies the raw material identity and ${Ai}_{m}$ denotes the additional information about the pharma raw material. Suppliers encrypt the $R_{1}$ using an encryption key ($k_{1}$) which is generated by symmetric encryption (AES), the generated cipher text ($T_{c1}$) is $T_{c1}\;\leftarrow \;E\left({k_{1},R}_{1}\right)$. Now, suppliers' materials are protected from malicious activities by generating a hash for the product provenance record R1 as shown in equation 10

(10) $$H\left(T_{c1}\right)\;\leftarrow \;{{PRk}_{si}\;\mathrm{mod}\;R}_{1}$$

$E_{sign1}$ ←$H\left(T_{c1}\right)$, where $E_{sign1}$ denotes the signature of $T_{c1}$ and $H\left(T_{c1}\right)$ is the hash of cipher text. To access the record $R_{1}$ drug manufactures require the suppliers' public key (${PUk}_{si}$) where i = 1. Algorithm 2 represents how smart contracts add products to suppliers, increasing stakeholder's visibility of existing stock products/items. A registered supplier smart contract is deployed once on the Ethereum blockchain and is responsible for maintaining track of the inventory system. If Suppliers want to add any product, they can specify the product_Name, product_Id, product_Quantity, and product_Price. Still, if the product already exists, the supplier makes the modifications on product_Quantity by entering the product_Id and updating the quantity sent to stakeholders to generate the event.

**Table:** 

Algorithm 2: Adding and Removing products to Suppliers
*Input*: product_Name, product_Id, product_Quantity and product_Price
*Output*: Products added to suppliers ($P_{s}$)
*Procedure*: Modifier ← onlyOwner (suppliers ($P_{s}$)) suppliers ($P_{s}$) add_product details in supplier smart contract
Restrict access to $P_{s}\;\epsilon \;S_{i}$
If supplier = registered and product_Id already exists then
Increase product_Quantity
Else add_product by setting product_Name, product_Quantity and product_Price
Endif
If select product_Id to remove then product_Removed ← product_Id
Else
No such product_Id existed
Endif
Revert smart contract state to false
Return number of products
Broadcast the updates by triggering the event
*EndProcedure*

Case2 Drug manufactures to distributor

Drug manufactures buy products from suppliers and sell finalized products to distributors. Algorithm 3 presents the process of selling products from drug manufactures to distributors. Initially, the smart contract verifies that both entities are registered, agrees to the next product sale, and pays the purchase price. If both conditions are satisfied, then the contract state changes to productRequestAgreed. If the conditions are unsatisfied, the contract state is changed to productRequestFailed.

**Table:** 

Algorithm 3: Distributor buy products from Drug manufactures
*Input*: Distributors Ethereum account address (${EA}_{di}$), Drug Manufactures Ethereum account address (${EA}_{dmi}$), product_Quantity and product_Price
*Output*: True/Flase
*Procedure*: Modifier ← onlyOwner ($P_{d}$)
Modifier ← onlyOwner ($P_{dm}$)
Contract state: buyfromDrugManufactures
Restrict access to $P_{d}\;\epsilon \;S_{i}$
If distributor agreed terms and product_Price = paid then
Contract state changed to productRequestAgreed
Create notification message and send to $P_{d}$
Else
Contract state changed to productRequesrFailed
Create notification message request failure
Endif
Revert smart contract state to false
Return number of products
Broadcast the updates by triggering the event
*EndProcedure*

Case3 Distributors and Drug Manufacture to Retailer

The retailer received the pharma products from either the distributor or drug manufactures and verified the product provenance record $R_{2}$ validity computed and $R_{2}$ represented as equation 11

(11) $$R_{2}\;\leftarrow \;{{Id}_{r1}⊕Id}_{d1}⊕{Id}_{dm1}⊕{Ai}_{m2}$$

Where ${Id}_{r1}$ represents the identity of a retailer, ${Id}_{d1}$ specifies the identity of the distributor, ${Id}_{dm1}$ represents the identity of the drug manufacture and ${Ai}_{m2}$ represents the additional information about the pharma raw material. Distributors or drug manufactures encrypt the $R_{2}$ using an encryption key ($k_{2}$) which is generated by symmetric encryption, the generated cipher text ($T_{c2}$) is $T_{c2}\;\leftarrow \;E\left({k_{2},R}_{2}\right)$. Now, retailers are protected from malicious activities by generating a hash for the product provenance record $R_{2}$ as as shown in equation 12

(12) $$H\left(T_{c2}\right)\;\leftarrow \;{{Sk}_{si}\;\mathrm{mod}\;R}_{2}$$

**Fig. 3:**
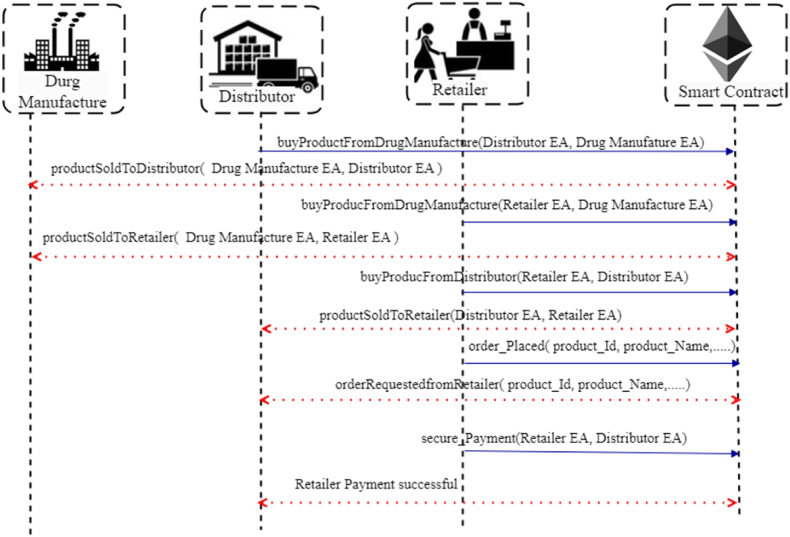
Sequence diagram showing the interaction between drug manufactures, distributor, retailer and smart contract.

$E_{sign2}$ ←$H\left(T_{c2}\right)$, where $E_{sign2}$ denotes the signature of $T_{c2}$. In algorithm 4, ordered products are shipped from distributor/drug manufactures to retailers. Initially, the smart contract verifies whether the product sale is agreed upon and payment has been successful, and then the smart contract states changes to saleRequestAgreed. If neither condition is met, the smart contract state is changed to saleRequestDenied. Fig. 3 illustrates the sequence flow of the drug manufactures, distributors and retailers with smart contracts.

**Table:** 

Algorithm 4: Distributor and Drug Manufacture to Retailer
*Input*: Distributors Ethereum account address (${EA}_{di}$), Drug manufactures Ethereum account address (${EA}_{dmi}$), dateof_Manufacture, and product_Quantity,
*Output*: True/Flase
*Procedure*: Modifier ← onlyOwner ($P_{d}$)
Modifier ← onlyOwner ($P_{dm}$)
Modifier ← onlyOwner ($P_{r}$)
Contract state: productsoldtoDistributor/DrugManufacures
Retailer state: readytoPurchase
Restrict access to $P_{r}\;\epsilon \;S_{i}$
If sale = agreed and payment (Algorithm 6) = successful then
Contract state changed to saleRequestAgreed
Emit success message
Else
Contract state changed to saleRequestDenied
Emit sale request failure message
Endif
Revert smart contract state to false
Broadcast the updates by triggering the event
*EndProcedure*

Case4 Distributor and Retailer to Consumer

**Fig. 4:**
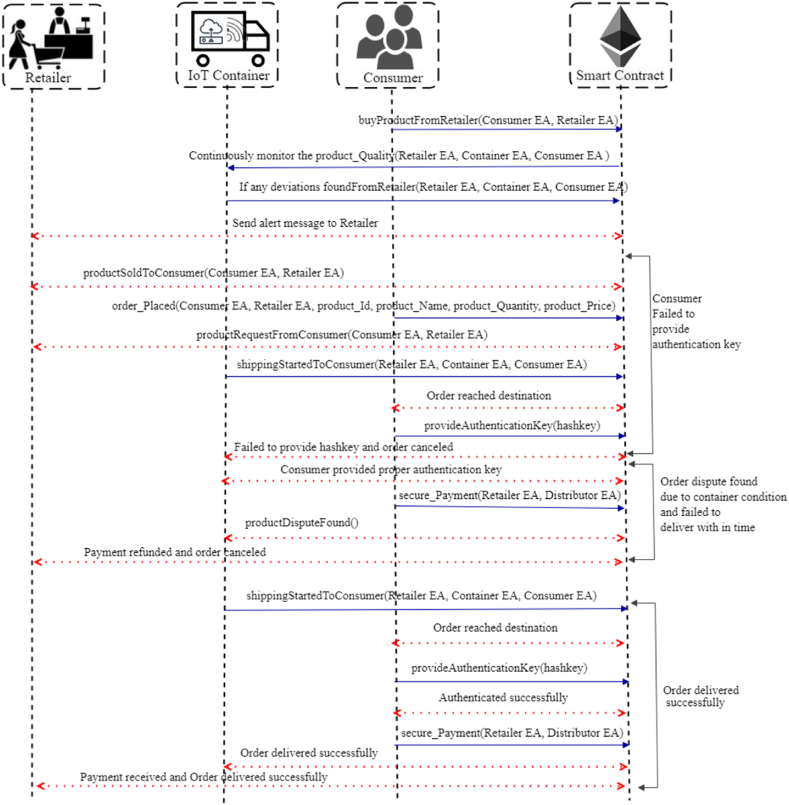
Sequence diagram showing the interaction between retailer, consumer and smart contract.

Finally, the consumers buy the pharma products from retailers or distributors. Algorithm 5 presents the complete selling process from distributors/retailers. Initially, the smart contract checks whether payment is successful; if payment is successful, the contract state changes to productSoldtoCustomer; otherwise, it emits productSaleDenied. If the product is delivered on time, the contract states changes to productDeliveredOnTime; otherwise, return productDelivereryFailedOnTime. In the cases of dispute found due to container conditions and in-time delivery, authentication failed due to the appropriate hash key and successful delivery shown in Fig. 4. If the consumer fails to provide the proper secret code during product dispatch, a smart contract offers a 24-h time frame to provide the correct secret code; if the period exceeds, half the amount would be refunded to the consumer, and product delivery would be terminated. In each case, stakeholders $S_{i}$ have to compute record $R_{i}$ and generate cipher text $T_{cti}$ to send the products to another stakeholder, they utilize the ${PRk}_{si}$ to generate signatures and provenance records. At the end of each case, a transaction is created. To verify the validity of the provenance record by following the equation

(13) $$E\left(E_{signi},G_{i}\right)=E\left(H\left(T_{ct1}⊕T_{ct2}\right)\right)$$

**Table:** 

Algorithm 5: Product Delivery Distributor and Retailer to Consumers
*Input*: Retailer Ethereum account address (${EA}_{ri}$), Distributor Ethereum account address (${EA}_{di}$), Customer Ethereum account address (${EA}_{ci}$), purchased_Date, product_Id, hashkey, current_Time, and delivery_Time
*Output*: Product delivered successfully
*Procedure*: Modifier ← onlyOwner ($P_{d}$)
Modifier ← onlyOwner ($P_{r}$)
Modifier ← onlyOwner ($P_{c}$)
Contract state: productReadytoBuy
Restrict access to $P_{c}\;\epsilon \;S_{i}$
If product_Payment (Algorithm 6) = successful then//to check payment
Contract state changed to productSoldtoCustomer
Emit purchase success message
Else
Contract state changed to productSaleDenied
Emit purchase failure message
Endif
If current_Time < delivery_Time then//exceed delivery time
Contract state changed to productDeliveredOnTime
Emit order cancellation message
Else
Refund payment to stakeholder ($S_{i}$)
Contract state changed to productDelivereryFailedOnTime
Revert smart contract state to false
Endif
Broadcast the updates by triggering the event
EndProcedure

### 4.3 Secure payment smart contract

In BPSCM, the stakeholders settle the payment for raw materials and products delivered to various stakeholders. Stakeholders have established secure payment through smart contracts. In algorithm 6, customers are verified through the kecchak256 hash function, and upon the hashed key match with the stored hash, the contract state is changed to customerVerificationSuccess. The payment has begun, and the balance is sufficient to buy the product. Then, transfer the total calculated amount to stakeholders and return paymentSuccess contract state. If the stakeholder balance is insufficient, the payment fails and returns an insufficientBalance contract state. In BPSCM, the stakeholders S_i_ have invoked a secured payment smart contract. The consumer pays the stakeholder S_i+1_ for successful product delivery, and the expense is expressed as S_E_. If the product delivery fails due to manufacturing or other issues, stakeholder S_i_ will refund stakeholder S_i+1_, representing the refund as S_R_.

**Table:** 

Algorithm 6: Secure Payment among Stakeholders
*Input*: product_Price, product_Id, stakeholders Ethereum account address ${EA}_{si}$ , where iϵ1,2,3,4
*Output*: Payment successfully
*Procedure*: Modifier ← onlyOwner ($S_{i}$)
ontract state: ReadytoPayment
Restrict access to $P_{c}\;\epsilon \;S_{i}$
Compute keccak256 hash key//customer verification process
Z ← keccak256(key)
If z = keyhash and check record provenance success using equation (13)
Contract state changed to customerVerificationSuccess
Start the payment process
Total = Calculate the no.of.products ∗ product_Price + transport_Fee
If check balance is to pay total
Transfer total payment to stakeholder ($S_{i}$)
Contract state changed to paymentSuccess
Emit product payment successful
Else
Balance is not sufficient to pay
Contract state changed to insufficientBalance
Endif
Else
Contract state changed to paymentFailure
Emit payment failure message
Endif
Broadcast the updates by triggering the event
*EndProcedure*

Our model is robust enough to track, monitor, and verify product consistency during various situations. Smart contracts track the IoT sensors' data deviation and trigger alert messages, especially for temperature and pressure. Smart contracts ensure all transactions are tamper-proof within BPSCM. A secure encryption schema maintains the confidentiality of the product's provenance record and prevents its information from being modified illegally throughout its circulation. Use the hash value to verify the legitimacy of provenance records without disclosing their origins to investigators.

Ethereum 2.0 with PoS improves key performance indicators in the PSCM system, including scalability, security, and energy efficiency. PoS improves scalability by improving transaction throughput, which is essential for managing substantial data and interactions in PSCM. It also decreases latency by allowing faster block validation and transaction confirmation times. PoS strengthens security by making 51 % of attacks less practical, as holding the majority of staked tokens is expensive. Furthermore, PoS minimizes processing expenses and energy usage, resulting in lower gas fees and a more cost-effective system for all participants. Key aspects to consider are throughput, latency, gas consumption, and security against attacks [54].

## 5 Testing and validation

The candidate BPSCM model simulation was run on five systems equipped with an Intel i7 CPU, 16 GB of RAM, and 1 TB of SSD capacity, running the Ubuntu 20.04 operating system and other necessary specifications to develop the entire architecture. These systems were designed explicitly as a peer-to-peer network of various entities (i.e., $P_{s},P_{r}$, $P_{dm}$, $P_{d}$, $P_{c}$). Specifically, we use the local Ethereum 2.0, Ganache, Geth client, Remix, and Metamask to determine the effectiveness and sustainability of smart contracts intended for all operations and the supply chain [11]. The included PoS, known for its energy efficiency, ensures miners do not require complex mathematical computations to validate transactions. After deploying the smart contracts, byte code is generated, and blocks are validated through PoS consensus. This process ensures a fair distribution of block creation and validation of the blocks upon PoS [48], instilling confidence in the technology's reliability. The smart contracts, designed with user security in mind, use modifiers to ensure that only users with the stakeholder role can access them. The smart contract's state is meticulously checked before the algorithm's execution and updated to the next state upon completion. Users create events to alert modifications and verify the results by examining the transaction-specific logs stored in the application development environment. These logs contain detailed information about the transaction output, events, gas usage amount, and exceptions. The ability to identify errors in the IDE by debugging the deployed code further enhances the sense of security in the system.

**Fig. 5:**
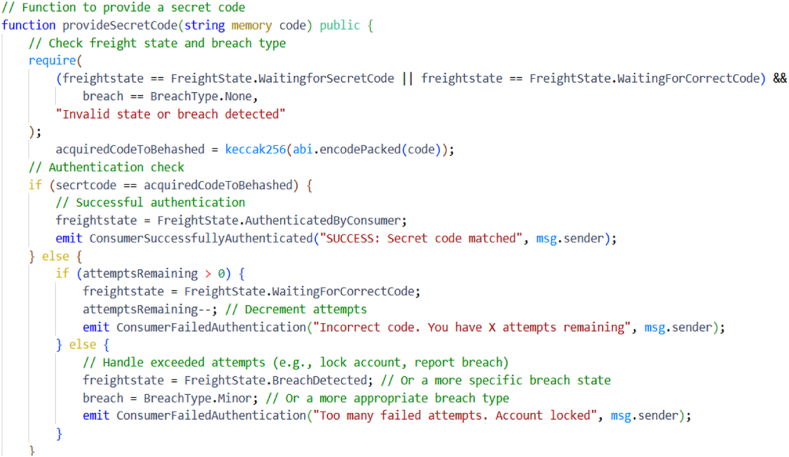
Smart contract code snippet for authenticity.

Moreover, the development environment offers transaction gas restrictions that may also be changed by the developer, offering an accurate representation of the mainnet network in Ethereum. The pharma supply chain, a critical sector, grapples with significant social and economic concerns, physical security, and freight delivery and quality difficulties. The high cost of these challenges is a significant barrier to achieving rapid evolution [31], underscoring the urgency and importance of finding effective solutions. The sample code for authenticity is shown in Fig. 5.

**Fig. 6:**
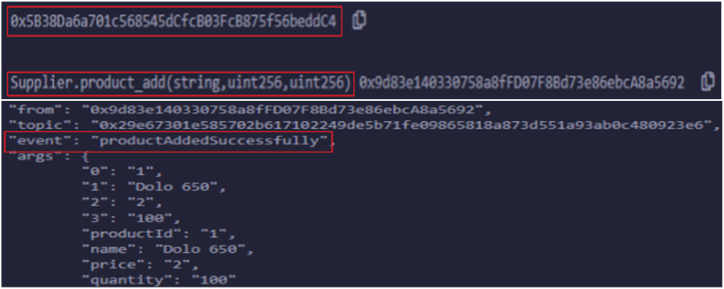
Logs triggered upon submitting supplier to add products.

In our testing scenario, all the smart contracts (Registration, Product Circulation, and Secure Payment) are deployed in the private Ethereum test network. Initially, all the stakeholders registered in the registration smart contract were assigned unique Ethereum addresses, and the 20-byte identifier was used to identify each stakeholder. The unique addresses of deployed contracts about diverse stakeholders are furnished in Table 4, along with gas consumption, transaction cost, and execution cost. Login() functionality extends seamlessly to all registered stakeholders without relying on their assigned roles. Upon the successful execution of the function without any errors, trigger an event notification to acknowledge smooth login completion. For instance, product_add() functionality is used by the supplier and drug_manufaturer to either include the product details or view them. Here, in Fig. 6, the snapshot shows the details of the successful inclusion of the product by the supplier.

**Table 4:** Deployed smart contracts with a unique address.

Contract name	Ethereum account address	Gas	Transaction cost	Execution cost
Suppliers	0 × 5B38Da6a701c568545dCfcB03FcB875f56beddC4	1381041	1201239	1069053
Drug manufactures	0xAb8483F64d9C6d1EcF9b849Ae677dD3315835cb2	1309535	1139054	1010690
Distributors	0 × 4B20993Bc481177ec7E8f571ceCaE8A9e22C02db	161209	140181	118697
Retailer	0x78731D3Ca6b7E34aC0F824c42a7cC18A495cabaB	1309494	1139018	1010698
Customer/Consumer	0x617F2E2fD72FD9D5503197092aC168c91465E7f2	1539385	1338969	1197987

**Fig. 7:**
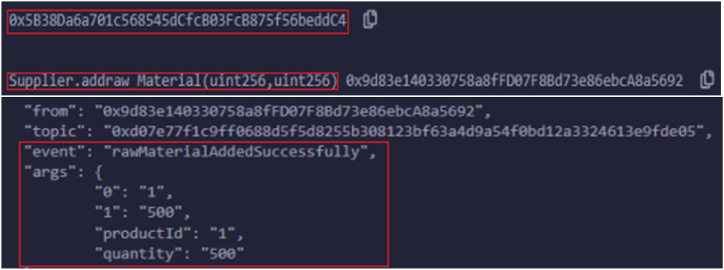
Logs triggered upon submitting supplier to add raw material.

If suppliers want to add any raw materials to facilitate the addition of new raw materials by suppliers to polish the pharma products, then the addraw_Material(1) function is invoked. This includes specific details such as product name and price. If this scenario is successful, the contract state transitions to rawMaterialAddedSuccessfully, and Fig. 7 visualizes the function execution logs to provide transparency into the raw material process.

**Fig. 8:**
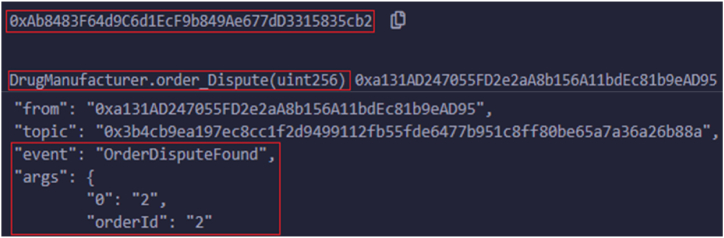
Logs triggered upon submitting drug manufacture find order dispute.

**Fig. 9:**
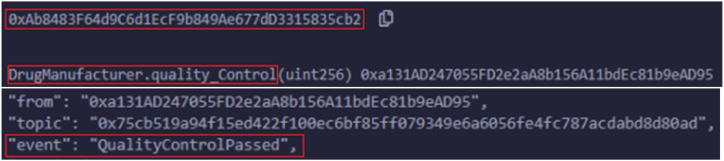
Logs triggered upon submitting drug manufacture to check product quality.

**Fig. 10:**
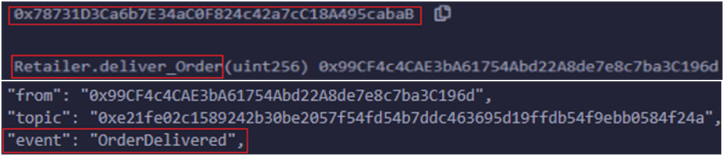
Logs triggered upon submitting retailers order delivery.

The remainder of the accounts belong to suppliers who keep adding their products by using unique item numbers with available quantities. These events notify the vendors by setting reactions if any changes to the inventory. In the same way, another real-world entity, drug manufacturers, identify any disputes that happen in products, which leads to the OrderDisputeFound state. Fig. 8 shows the logs related to order disputes. The order dispute may occur due to the need to provide a proper authentication key, IoT container conditions, and delays in time. The product quality is checked in each phase to deliver the item; the product quality depends on the date of manufacturing medicines, the molecules involved, and the drug manufacturing company. If there is no compromise in product quality, then the state transitions to QualityControlPassed. Fig. 9 shows the related logs for handling the distribution of products by distributors and retailers. After conducting a quality inspection, distributors successfully supply goods to retailers, distributing the final products to customers. Once all quality checks are completed, merchants deliver the products to consumers. Fig. 10 illustrates the successful delivery of orders by merchants.

### 5.1 Security analysis

**Fig. 11:**
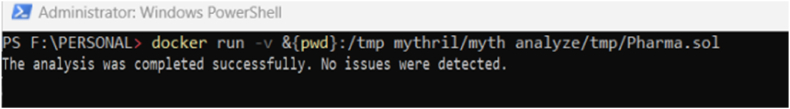
Smart contract Vulnerability analysis report using MyThril tool.

This section examines potential vulnerabilities and security concerns in smart contracts within the proposed pharma supply chain solution. The presented model demonstrates its ability to ensure trust, data integrity, availability, non-repudiation, and cyber-attack resilience. Like any other complicated system, the Ethereum blockchain is vulnerable to various security concerns, including network and consensus attacks. Network attackers use DDoS and Sybil attacks to impede operations by exploiting communication flaws. Attackers using consensus techniques aim to compromise the fundamental validation procedure by attempting to manipulate transactions via majority control. Although research [43] highlights the promising solutions to Ethereum's security concerns, attention is still needed. Proactive security measures and constant assessment are required due to the growing scale of the network, the distribution of miners, and changing attack methods. Recognizing Ethereum's advantages and the constantly evolving possibility landscape enables developers, consumers, and other stakeholders to make well-informed decisions within this continually changing technical ecosystem. MyThril, an open-source program known for its outstanding performance in identifying security flaws in smart contracts, is utilized by the presented applications. MyThril is written in Python and executed using a command line tool [40]. The security analysis relies on contaminate analysis, dynamic symbolic execution, and control flow verification to examine the Ethereum Virtual Machine (EVM) bytecode for vulnerabilities. A Docker image of the tool was used to test smart contract vulnerability. The program carefully inspected files for flaws such as unchecked return values, assert violations, unprotected Ether withdrawals, delegate calls to untrusted callees, integer underflows, unauthorized storage writes, etc. [41,42]. The audit of the pharma contract produced a comforting result, as shown in Fig. 11. The analysis was completed successfully, and no issues were detected. These results reinforce our system's credibility by offering solid proof of the security of our suggested smart contract-based pharma supply chain.

However, timestamp dependencies (smart contracts that rely on timestamps are vulnerable to tampering, jeopardizing their functioning or security), transaction ordering dependencies (they arise when the result of one transaction depends on the sequence in which other transactions occur), integer overflows and underflows leads to contract's behavior may become unpredictable and susceptible to manipulation. The re-entrancy attack happens when a malicious contract actively calls back into the target contract before the first transaction is complete. This hole enables the attacker to repeatedly call the vulnerable function, draining funds before the contract can update its internal state. The experiment used acknowledged best practices for smart contract development to mitigate this. Specifically, we structured our smart contracts to conduct state changes before making any external calls, removing the possibility of external contracts violating re-entrancy flaws. Furthermore, modifiers to guarantee that the execution process is locked during crucial operations, eliminating recursive call loops that might jeopardize the contract's state integrity. Moreover, we used the Oyente tool, which is well-known for its in-depth analytical capabilities, for a more extensive vulnerability evaluation.

Oyente carefully examined the code to identify various EVM security flaws [44]. This comprehensive analysis resulted in a report summarizing EVM coverage with 60.2 %, which means Oyente significantly analyzed byte code instructions more than 50 % and a critical "false" decision for all reported vulnerabilities, indicating high security in our code, as shown in Table 5. Oyente is compatible with several older versions of the Solidity compiler, which is crucial for understanding. As a result, before the study, code syntax changes were required to comply with these restrictions. Moreover, Oyente offers insightful direction and line-by-line suggestions for fixing any possible flaws, enabling iterative improvement and optimization of our code. This meticulous process, which included intelligent error detection and extensive security research, gave us confidence in the resilience and security of our smart contract usage.

**Table 5:** Smart contract Vulnerability analysis report using Oyente tool.

Parameter	Results
EVM Code Coverage	60.2 %
Integer Underflow	False
Integer Overflow	False
Timestamp Dependency	False
Re-entrancy Vulnerability	False
Transaction ordering dependency (TOD)	False
Parity multi-sig Bug	False

The BPSCM uses smart contract capabilities to manage authentication and access control, with restricted modifiers allowing registered stakeholders to execute operations. In addition, the system hashed every product detail with a timestamp. The EdDSA version we used in BPSCM is unforgivable by MITM (Man-in-the-Middle). Thus, the system prevents **Replay** and **MITM** attacks. Since only the legitimate user can access the private key, the attacker cannot access the product, even if he replaces it with his own EA and public key. Therefore, the system would reject the transaction and reverse the contract state if an attacker were to impersonate a legitimate user's IP address or EA address. **Integrity** ensures no one alters data to exclude key information by using logs and creating events. Our model can monitor and trace the history of product details. The Ethereum address of the function call initiator is always captured and included in the logs, and **non-repudiation** confirms the stakeholder's identity. It guarantees that the user cannot retract their actions. The smart contract functions are executed, and logs are available to stakeholders to ensure their availability. It provides all services are available to users and protects against **Denial of Service** (DoS) due to an immutable public ledger.

The publicly available private Ethereum blockchain was chosen for BPSCM because it offers anonymity to all stakeholders, even though private blockchains like Multichain, Hyperledger, and private Ethereum offer transaction encryption and restricted access control. It provides pseudonymity, and the blockchain's unchangeable record of timestamped transactions encourages supply chain accountability and transparency. BPSCB ensures the **confidentiality** by using symmetric encryption. This openness makes it possible for all parties to be informed about inventory levels and makes it easier to respond quickly to unforeseen changes in demand. IPFS hash stores product information to reduce the storage burden. The product provenance record is generated through unique stakeholder identification with XoR operations and broadcasted to the network. Initially, we encrypted the raw material using symmetric encryption and performed record verification through EdDSA. It is difficult for adversaries to tamper with product information. This ensures that our model BPSCM is resistant to **tampering attacks**. A **key guessing attack** is similar to a brute-force attack, which involves a cryptanalyst attempting all possible guesses for the private key. The suggested approach generates 256-bit long private keys using the SHA-256 hash algorithm. Hence, a brute force assault cannot compromise the security of the proposed system in a finite amount of time [56].

As previously stated, the product provenance record would be hashed into 256-bit codes to ensure authenticity during pharma product circulation. It guarantees the product's traceability and prevents unwanted entities from forbidden alterations. As a result, not all stakeholders can change or retrieve the hash value. Thus, our BPSCM successfully threw the **collusion attack**. If external adversaries succeed, password-guessing attacks can compromise stakeholder identities. We generate high-security identities that utilize the random verification number and hash of private keys and leverage smart contracts for secure registration. This approach enhances security against **impersonation attacks**. Table 6 compares the security analysis of the existing model regarding the perception of the above content.

**Table 6:** Comparision of security analysis with state-of art techniques.

Security Property	[30]	[31]	[33]	[56]	Proposed model
Collusion attack	Ć	×	×	Ć	Ć
Tampering attack	Ć	×	×	Ć	Ć
Key guessing attack	Ć	Ć	Ć	Ć	Ć
Impersonation attack	×	×	×	×	Ć
DDoS	×	×	×	×	Ć
Re-entrancy attack	×	×	×	×	Ć

The presented approach uses protocols to ensure only legitimate parties validate the transaction and block creation using robust authentication implemented in the registration phase. Hence, the loophole for the possibility of triggering 51 % of attacks was prevented to a greater extent. Additionally, Ethereum 2.0's PoS protocol provides substantial benefits for securing a BPSCM system. Unlike PoW, PoS is based on economic incentives, making it financially difficult for attackers to take control. Also, reducing penalties prevents malicious behaviour by penalizing validators who attempt to disrupt the network. In addition, PoS features like checkpointing and a safety oracle improve security by restricting the time frame for blockchain reorganization and identifying discrepancies [54]. These combined considerations make a 51 % attack on Ethereum 2.0 exceedingly difficult and economically unfeasible in the context of a PSCM. The decentralized structure of PoS and the financial disincentives for malicious behaviour offers a strong and secure foundation for securing sensitive pharmaceutical data and preserving supply integrity.

## 6 Performance evaluation

**Fig. 12:**
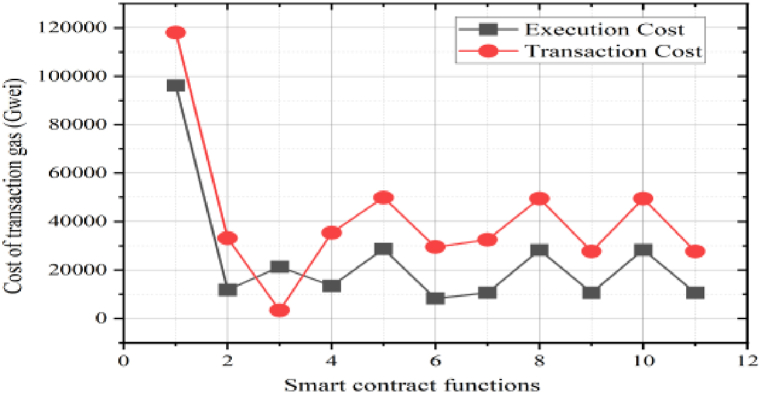
Performance analysis of Pharma smart contracts.

This section evaluates the proposed scheme's effectiveness and efficiency through various performance metrics. This assessment employs gas consumption, throughput, latency, and computational cost. When submitted to the private Ethereum blockchain, a minimum gas price is needed to cover a transaction's processing costs. Conveniently, Remix IDE's output interface estimates this cost for every transaction. Fig. 12 lists the expenses for the different smart contract code functions. As shown in the black line, the computing requirement of invoked operations indicates execution costs. Higher execution costs are a natural result of more sophisticated functions. Conversely, the red line represents transaction costs, including deploying the complete smart contract code into the blockchain. This is independent of code size and function, in contrast to execution cost. Manufacturers can optimize smart contracts cost-effectively and efficiently by identifying these different expenses [31,32].

### 6.1 Scalability

**Fig. 13:**
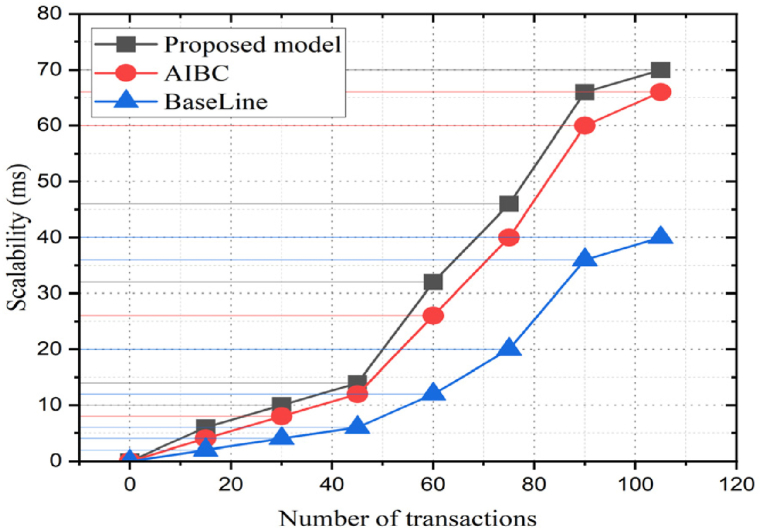
Scalability analysis.

Scalability is crucial when implementing blockchain in PSCM, mainly due to the large volume of data generated. However, to enhance scalability, layer-2 solutions, namely, state channels or sidechains (to allow off-chain transactions and reduce the burden on the main blockchain) and sharding (splits a blockchain network into smaller partitions called shards to process more transactions), are implemented [61]. In our BPSCM, only approved participants can validate transactions, significantly improving the network performance. The proposed framework utilizes IPFS to store the hashed data instead of raw data in the immutable ledger. Due to hash values, the data fetches quickly, improving blockchain response time. Fig. 13 provides precise information regarding the blockchain based on IPFS, which is more scalable than the traditional baseline models AIBC [45].

### 6.2 Computation cost

**Fig. 14:**
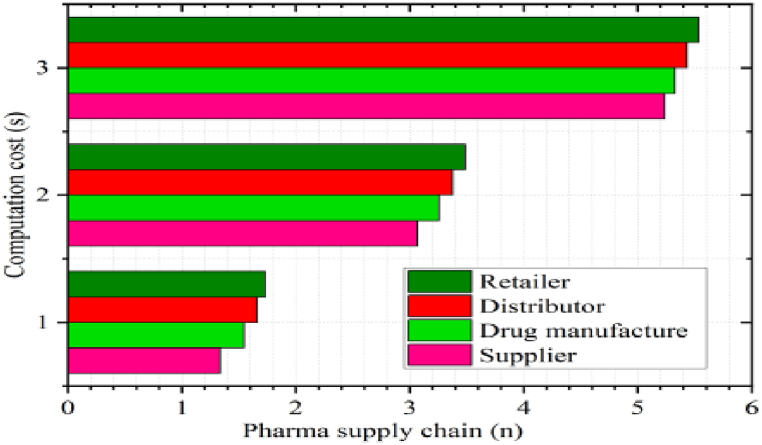
Computation cost analysis.

To evaluate the computation cost of the validation process in a real-world environment, we mainly focus on encrypting the product details using a symmetric algorithm, hashing and EdDSA to verify user integrity and product verification [30]. In our framework, validation is carried out through suppliers, distributors, retailers, and drug manufacturers. Fig. 14 demonstrates the effectiveness of our approach, assuming a distinct number of supply chains (each with a supplier, distributor, retailer, and drug manufacturer) concerning computation cost. For instance, authenticating a single product record takes 1.4 s, takes 1.5 s, takes 1.6 s, and Pr takes 1.8 s for one single supply chain. Our BPSCM offers affordable computing costs and applicability for various use cases [28].

### 6.3 Throughput

Smart contracts can be deployed, executed, and invoked at different rates across blockchain platforms. Thus, it is vital to monitor transaction throughput, typically calculated as the rate at which the blockchain network conducts legitimate transactions during a certain period. The throughput shows the number of transactions completed per second, which is calculated using equation (14) [51].

(14) $$T=\frac{C\left(T_{tx}\;in\;\left(T_{b},T_{i}\right)\right)}{T_{b}-T_{i}}$$

**Fig. 15:**
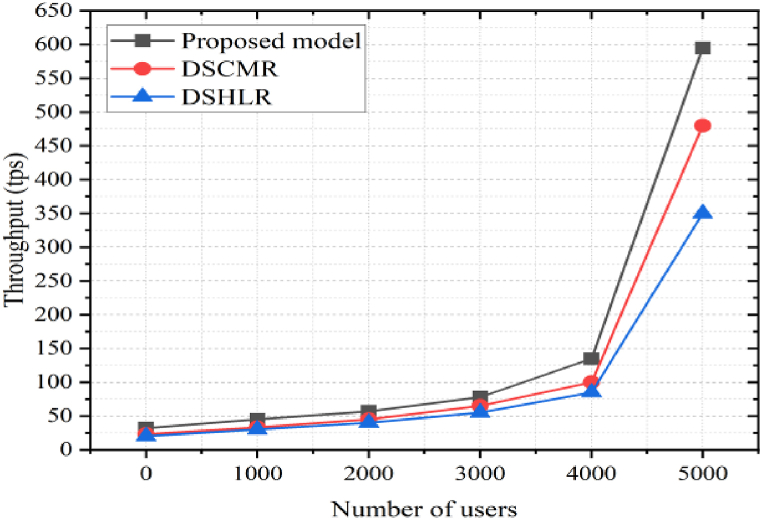
Throughput analysis.

Where, T is transaction throughput, C is count, $T_{tx}$ is the total number of submitted transactions, $T_{b}$ is block confirmation time, $T_{i}$ transaction submission time. Fig. 15 shows that the transaction initiation time is constant regardless of the number of users. We vary the number of users from 1000 to 5000 until 4000; throughput variation is much less at 5000 users. Throughput drastically increases for the proposed and existing models. It also shows that our approach performs better than the existing models DSCMR [22] and DSHLR [46].

### 6.4 Latency

The system requires a certain period to validate a transaction before it is sent to the network. Transaction latency is when a transaction is entered, confirmed, and committed, and the result is made available to every user on the network. In BPSCM, various factors impact latency, including block generation time, network propagation delay, and transaction validation time. Our solution is designed to minimize latency through efficient block formation and transaction processing processes. This statistic is measured for each transaction in terms of seconds and expressed as equation (15) [51].

(15) $$L=\frac{\sum T_{tx}\left(T_{confirm}-T_{input}\right)}{C(T_{tx}\;in\;\left(\left(T_{b},T_{i}\right)\right)}$$

**Fig. 16:**
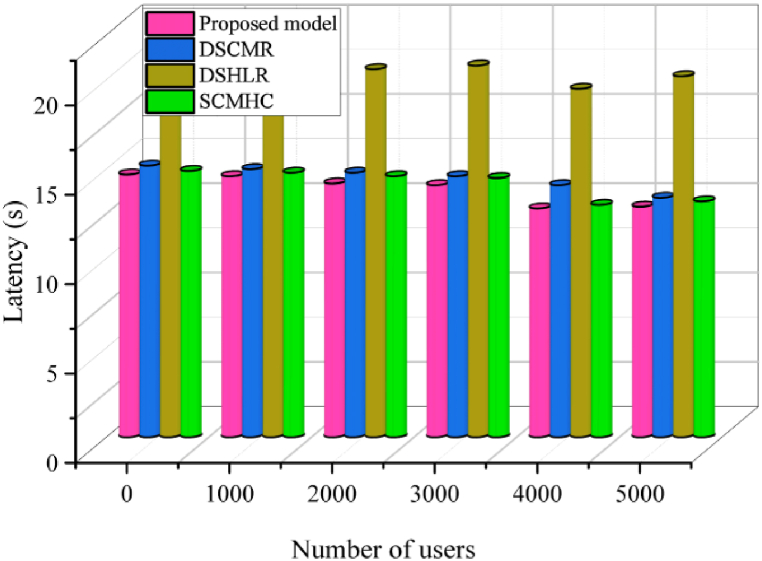
Latency analysis to invoke the transactions.

**Fig. 17:**
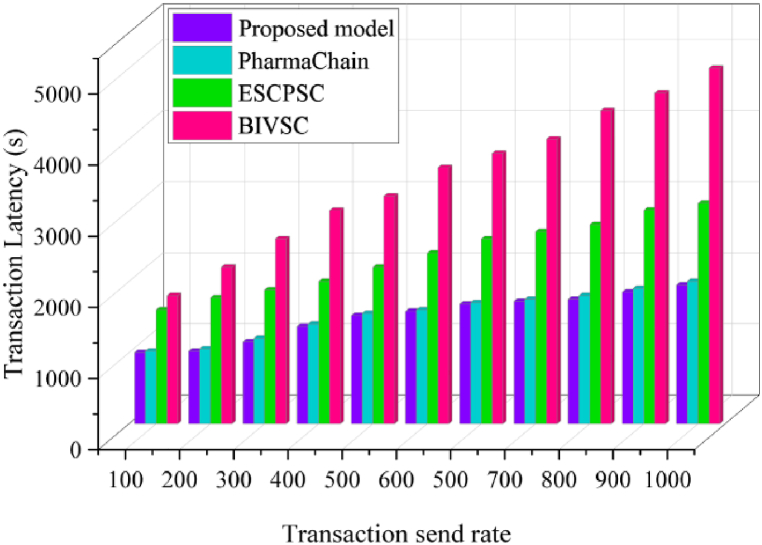
Transaction latency analysis.

Where, L is latency, $T_{confirm}$ is transaction confirmation and $T_{input}$ is transaction input. The proposed BPSCM model for the pharmaceutical supply chain outperforms compared to BIVSC [47], PharmaChain [11] and ESCPSC [31] in terms of average processing time. Fig. 17 highlights this benefit in terms of performance, whereby by increasing the number of transactions for shipping products, the average transaction latency is low compared to existing models. For 100 average number of transactions, it records the low transaction delay compared to existing models [11,31,47]. After that, the latency grows linearly, although it still has a significant advantage over the examined models. However, this demonstrates the enhanced effectiveness of our proposed framework with varying numbers of transaction requests. On the other hand, to prove the effectiveness of our BPSCM approach, we considered latency analysis to invoke transaction efficiency by changing the number of users from 1000 to 5000. in this case, our strategy also yields enhanced improvement compared to existing models SCMHC [19], DSCMR [22], and DSHLR [46], as shown in Fig. 16, Fig. 17.

### 6.5 Qualitative comparison

Table 7 summarizes the test results alongside those of other relevant solutions. This comparison aims to showcase the proposed solution's advantages within the pharmaceutical supply chain. The efforts undertaken in this work ensure the reliability and security of the proposed solution for the intended task. The suggested BPSCM decentralized approach eliminates the requirement for extra offline storage compared to the frameworks presented in Refs. [[28], [29], [30], [31],[33], [34], [35], [36], [37], [38], [39], [40], [41], [42], [43], [44], [45], [46], [47]]. Additionally, it offers DApps compatibility, a feature that must be added to existing solutions. This DApps compatibility fosters product sharing and reduces the risk of single points of failure. Additionally, the network operates independently, eliminating reliance on external entities like government agencies or developers for transaction validation.

**Table 7:** Comparison to the cutting-edge technology.

Ref	Security Analysis using tool	Cryptography Technique	Storage	BC Platform	Cost analysis	Dapp
[28]	**×**	**×**	**×**	Ć	Ć	**×**
[29]	**×**	**×**	**×**	Ć	NA	Ć
[30]	Ć	Ć	**×**	Ć	Ć	**×**
[31]	**×**	**×**	**×**	Ć	Ć	**×**
[32]	**×**	**×**	Ć	Ć	Ć	Ć
[33]	**×**	**×**	**×**	Ć	**×**	Ć
[35]	Ć	Ć	**×**	Ć	**×**	**×**
[36]	Ć	Ć	**×**	Ć	NA	**×**
[37]	**×**	**×**	**×**	Ć	Ć	Ć
Our Work	Ć	Ć	Ć	Ć	Ć	Ć

## 7 Conclusion

This paper presented a novel blockchain-based PSCM framework that addresses the critical challenges of privacy, authentication, and data provenance in traditional online systems. The proposed solution leverages smart contracts and cryptographic mechanisms to ensure secure and transparent tracking of pharmaceutical products throughout the supply chain lifecycle. Real-world entities are integrated into the framework, enabling real-time monitoring and automated alerts for potential quality deviations. The proposed BPSCM is implemented in three phases: registration, product circulation, and secured smart payment contracts. For security enhancement, EdDSA and symmetric encryption have been included for product provenance record verification. Security analysis demonstrates that the framework can withstand various cyber-attacks, and smart contract vulnerability analysis has been established. Finally, in terms of efficacy, the tested results show improvements in the metrics of throughput, reduced latency, and computation cost compared to state-of-the-art models. In the future, we plan to integrate Artificial Intelligence techniques for drug recommendation and analysis. More focus on scalability issues and integration of cutting-edge technology.

## CRediT authorship contribution statement

**Adla Padma:** Writing – original draft, Software, Resources, Methodology, Conceptualization. **Mangayarkarasi Ramaiah:** Writing – review & editing, Validation, Investigation, Formal analysis.

## Data availability

Data will be made available on request.

## Declaration of competing interest

The authors declare that they have no known competing financial interests or personal relationships that could have appeared to influence the work reported in this paper.
